# Genotyping of Cytomegalovirus from Symptomatic Infected Neonates in Iraq

**DOI:** 10.4269/ajtmh.18-0152

**Published:** 2019-02-25

**Authors:** Sevan N. Alwan, Haidar A. Shamran, Avan H. Ghaib, Haider S. Kadhim, Qasim S. Al-Mayah, Atheer J. AL-Saffar, Ali H. Bayati, Hala S. Arif, Jianmin Fu, Brian L. Wickes

**Affiliations:** 1 Department of Biochemistry and Structural Biology, UT Health at San Antonio, San Antonio, Texas;; 2 Medical Research Unit, College of Medicine, University of AL-Nahrain, Baghdad, Iraq;; 3 Microbiology and Immunology Department, College of Medicine, University of Sulaimani, Sulaymaniyah, Iraq;; 4 Microbiology Department, College of Medicine, Al-Nahrain University, Baghdad, Iraq;; 5 Community and Family Medicine Department, College of Medicine, Al-Nahrain University, Baghdad, Iraq;; 6 Community Health Department, Technical College of Health, Sulaimani Polytechnic University, Sulaymaniyah, Kurdistan Region, Iraq;; 7 Pediatric Department, College of Medicine, Al-Nahrain University, Baghdad, Iraq;; 8 Department of Microbiology, Immunology, and Molecular Genetics, UT Heath at San Antonio, San Antonio, Texas

## Abstract

Among all other viruses, human cytomegalovirus (HCMV) is the most frequent cause of congenital infection worldwide. Strain variation in HCMV may predict severity or outcome of congenital HCMV disease. Previous studies have associated a particular genotype with specific sequelae or more severe illness, but the results were contradictory. There are no previous studies addressing the genotype of HCMV in Iraq. Therefore, the present study is aimed at molecular detection and genotyping of HCMV isolated from symptomatic congenitally/perinatally infected neonates. This prospective study comprised 24 serum samples from symptomatic neonates with congenital/perinatal infection. Viral DNA was extracted from these serum samples; nested polymerase chain reaction was used to amplify the HCMV gB (*UL55*) gene. Polymerase chain reaction products of the second round of amplification were subjected to direct Sanger sequencing. Bioedit and MEGA5 software (EMBL-EBI, Hinxton, Cambridgeshire, UK) were used for alignment and construction of a phylogenetic tree. Human cytomegalovirus DNA was detected in 23 of 24 samples (95.8%). According to the phylogenetic analysis, three genotypes of the virus were identified; gB1, gB2, and gB3 genotypes. However, the gB4 genotype was not detected. Human cytomegalovirus gB3 was the most frequent genotype: 14 of 24 (58.33%) among symptomatic infected infants, followed by gB1 (6/24; 25%) and gB2 (4/24; 16.67%). A mixed HCMV infection with gB3/gB1 was detected in only one case. Human cytomegalovirus gB3 was the most predominant genotype among symptomatic congenitally/perinatally HCMV-infected neonates. No association was found between B3 genotype and specific clinical presentation. Jaundice was the most common clinical feature among symptomatically infected neonates, followed by hepatosplenomegaly.

## INTRODUCTION

Human cytomegalovirus (HCMV) circulates worldwide and is endemic in the human population without seasonal variation.^1,2^ Like other herpesviruses, HCMV has the ability to establish latency in different types of cells.^3,4^ Human cytomegalovirus infection is the most frequent cause of congenital infection worldwide and is the leading cause of permanent disability and birth defects.^5,6^ Most children with congenital cytomegalovirus are born to cytomegalovirus-seropositive women. Human cytomegalovirus can be transmitted from pregnant women to her fetus through the placenta; this transmission can occur even in the setting of preexisting maternal immunity.^7,8^ Perinatal HCMV can also infect the newborn infant via ingestion of breast milk.^9^

Prematurely born infants with perinatal HCMV were found to be at higher risk for HCMV-associated diseases.^10^ Greater than 90% of symptomatic congenitally infected infants develop long-term neurological sequelae, impaired vision, and developmental disability.^11^ This incidence is far greater than that of the better known chromosomal disorder Down syndrome.^12^ On the other hand, approximately 10–15% of asymptomatic congenital HCMV infections will later develop long-term neurological sequelae.^11,13^ The seroprevalence of HCMV in developing countries is higher than that in developed countries.^2^ This observation is important for congenital HCMV epidemiology. The incidence of congenital infection is directly correlated with the seroprevalence of HCMV antibodies in the population.^14,15^

Women of childbearing age are at major risk of giving birth to infants with congenital infection if the infection is acquired during pregnancy.^16^ Unfortunately, six studies in Iraq reported a high prevalence of HCMV IgM in non-married women and pregnant women.^17^ Many factors amplify the magnitude of HCMV as a health problem, such as lack of specific antiviral therapy and licensed vaccine. Furthermore, most maternal and newborn infections are asymptomatic and, therefore, not recognized at birth.^18–20^

An effective vaccine to protect mothers against HCMV infection during pregnancy is urgently needed to reduce the burden of disease.^15,21,22^ A number of vaccine candidates are under investigation.^18,20^ Glycoprotein B is an important component of recombinant vaccines under trial; therefore, more studies on genetic variability data in Glycoprotein B (gB) gene may help to determine the optimal strains for vaccine development.^15^

gB is a type 1 transmembrane protein and represents a highly conserved class III fusion protein present in members of the Herpesviridae family.^23^ The gene that encodes gB (*gpUL55*) is located in the central region of unique long genes (*UL*) of the HCMV genome. There are four major gB genotypes, which are determined based on the region surrounding the proteolytic cleavage site,^24^ although additional genotypes have also been described.^25^ Genotyping of HCMV has mainly focused on envelope glycoproteins gB (*UL55*) and gH (*UL75*), which play a role in virus entry and are major targets for neutralizing antibody response.^26,27^

Recently, a study began to define target structures within gB that are recognized by virus-neutralizing antibodies.^28^ In addition, there is evidence indicating that the gB genotype of HCMV strains may influence the clinical outcome of acquired HCMV infection.^29,30^ Earlier studies have shown that gB1 genotype is associated with hepatosplenomegaly.^31,32^ A study from India suggested an association between gB2 genotype and manifestation of hearing impairment and symptoms of central nervous system diseases in congenitally and perinatally HCMV-infected infants.^30^ A study from Spain revealed that the gB2 genotype was associated with abnormal image findings by ultrasound and/or magnetic resonance in congenitally infected fetus and newborns, whereas gB4 might be associated with a lower risk of abnormal image findings.^33^ However, these findings need to be explored further. Previous studies have associated particular gB genotypes with severe congenital HCMV infection, but the information is contradictory between reports.^34–39^

Two studies from Iraq were recently carried out, aimed to fill the gap of information regarding the prevalence of HCMV infection in children in Middle Eastern countries^14,15^; the study result shows a high prevalence of HCMV infection among neonates with symptomatic congenital infections^40^ and also variation in the most predominant clinical signs compared with another Middle Eastern study among hospitalized symptomatic HCMV-infected children.^41^ This variation may be relevant to variation in HCMV strains. Therefore, the present study focused on molecular detection and genotyping of HCMV isolated from infected neonates, based on gB gene.

## MATERIALS AND METHODS

This study was performed on serum samples from 24 neonates diagnosed with congenital or perinatal HCMV infection obtained at the central laboratories of Children’s Welfare Hospital and Imamein Kadhimein Medical City, Baghdad, Iraq, from January 2015 to December 2015. All neonates were admitted to the neonatal intensive care unit at the Child Protection Teaching Hospital and Imamein Kadhimein Medical City. The age range of the neonates is between 1 and 30 days. Neonates admitted to the neonatal intensive care unit were selected by consecutive sampling inclusion criteria as follows: neonates with serum samples positive for HCMV IgM antibody and neonates exhibiting various symptoms of congenital/perinatal infection, such as jaundice, petechial rash, hepatosplenomegaly, pneumonitis, congenital heart diseases, and congenital malformations, especially those involving the central nervous system and ophthalmological abnormalities. Clinical manifestation was determined by consultation with a pediatric specialist and verification of the information in the medical record. Ethical approval to perform the study was obtained from the Research Ethical Committee at the College of Medicine, Al-Nahrain University.

Approximately 2 mL of venous blood was obtained from each neonate, and blood samples were allowed to clot at room temperature and then centrifuged to collect sera. Sera were stored at −20°C until testing. Human cytomegalovirus infection was identified as HCMV IgM antibody positive by using the electrochemiluminescence immunoassay (ECLIA) kit (Roche, Penzberg, Germany) according to the manufacturer’s instructions. Viral DNA was extracted from 200 μL serum and tested for the presence of HCMV by polymerase chain reaction (PCR) amplification. The amplification reaction was performed using nested PCR. The outer primer sequences for the first round of nested-PCR amplification were gB1246 (5′-GGAAACGTGTCCGTCTT-3′) and gB1724 (5′ GAGTAGCAGCGTCCTGGCGA-3′), with a predicted fragment length of 478 bp. The inner primers used for the second round were gB (5′-TGGAACTGGAACGTTTGGCC-3′) and gB1604 (5′-GAAACGCGCGCGGCAATCGG-3′) with a predicted fragment length of 305 bp.^42^ Polymerase chain reaction products were sequenced using cycle sequencing by ABI PRISM^®^ BigDye^™^ Terminator cycle sequencing kit v.3.0 (Applied Biosystem, Foster City, CA). The resultant sequences were aligned with eight HCMV gB sequences in GenBank (National Center for Biotechnology Information) representing the four known genotypes of the virus using Bioedit software. These were M60927.1 and M60929.1 for gB1; M60931.1 and M60932.1 for gB2; M60933.1 and M60934.1 for gB3; and M60926.1 and M60924.1 for gB4. MView software (EMBL-EBI, Hinxton, Cambridgeshire, UK; available at http://www.ebi.ac.uk/ Tools/msa/mview/) was used for this alignment. MEGA5 software (http://www.megasoftware.net) was used to construct a phylogenetic tree with 1,000 bootstrap replicates.

## RESULTS

**Figure 1.**
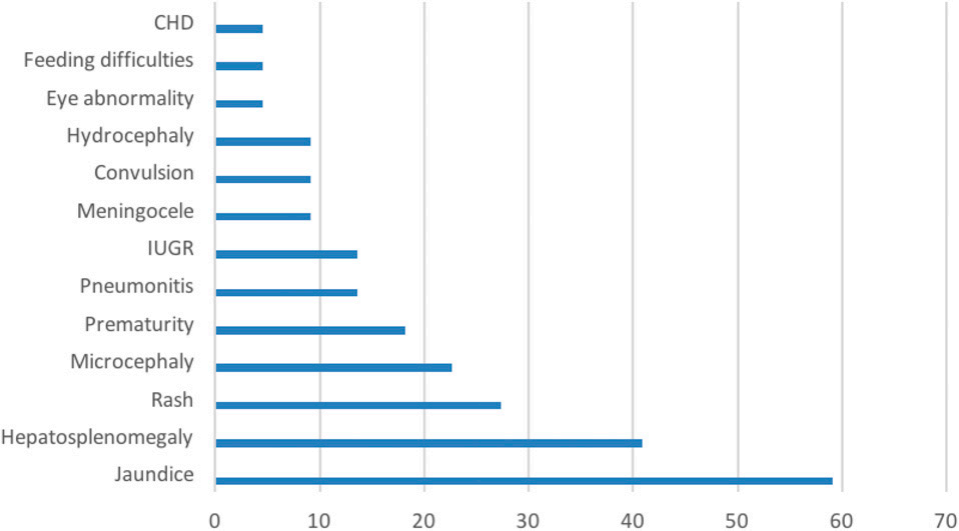
Clinical signs in human cytomegalovirus–infected neonates, listed in order of frequency. CHD = congenital heart diseases; IUGR = intrauterine growth restriction. This figure appears in color at www.ajtmh.org.

**Figure 2.**
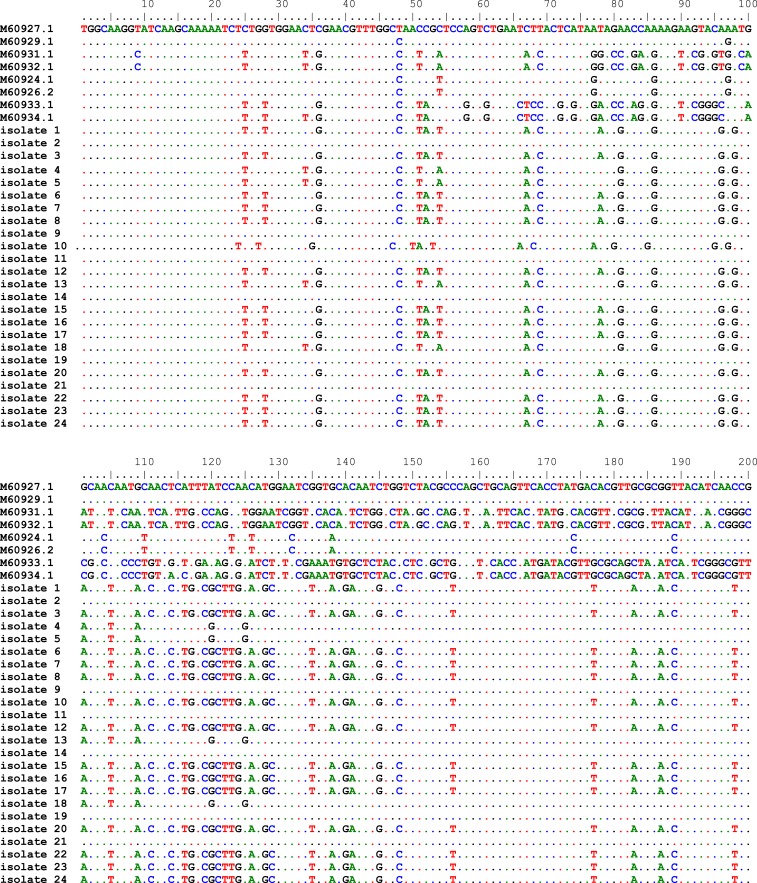

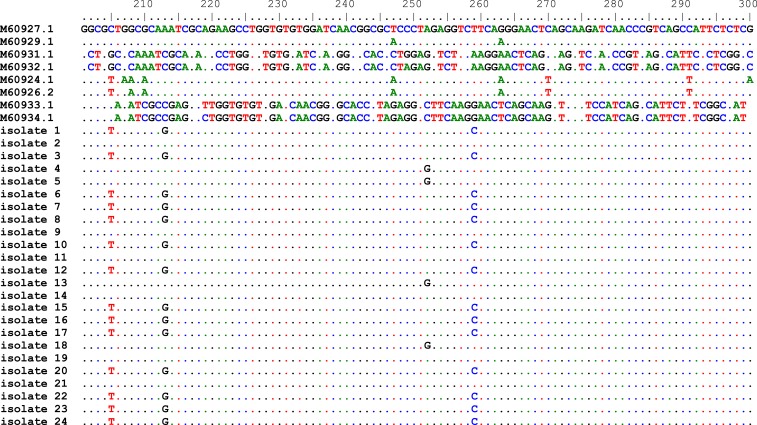
The alignment result of human cytomegalovirus sequences with the reference genotypes from the National Center for Biotechnology Information using Bioedit software. This figure appears in color at www.ajtmh.org.

Of the 23 symptomatic infected neonates, there was a higher prevalence rate among males (60.9%) than females (39.1%). The average age of HCMV detection was 13.8 days after birth and the second week of postpartum period was the medium age of HCMV detection as shown in Table 1. Three neonates (13%) were born with low birth weight and four (17.4%) were preterm neonates. Jaundice was the most common feature (59.1%) displayed by the symptomatic infected neonates, followed by hepatosplenomegaly (40.9%), rash (27.3%), and microcephaly (22.7%). Other clinical signs were less frequent (Figure 1). Twenty-four samples gave positive results for HCMV IgM antibody; DNA was successfully extracted from 23 sera. The strains were typed based on the DNA sequence of the gB (*UL55*) gene. The alignment of these sequences with eight reference sequences is shown in Figure 2.

**Table 1:** Clinical details and genotype of human cytomegalovirus–infected neonates

Neonate	Day of viral detection	Neonate with LBW	Neonate with SGA	Gender	Clinical manifestations	Genotype
1	3	–	–	Male	Jaundice, hepatosplenomegaly, and convulsion	B2
2	1	–	–	Male	Jaundice and hepatosplenomegaly	B3
3	18	–	–	Male	Hepatosplenomegaly and rash	B3
4	20	–	–	Male	Jaundice and microcephaly	B2
5	14	LBW	SGA	Female	Microcephaly and prematurity	B2
6	24	–	–	Male	Jaundice and hepatosplenomegaly	B3
7	7	–	–	Female	Edema of eyelid	B3
8	14	–	–	Male	Jaundice, hepatosplenomegaly, rash, and IUGR	B3 and B1
9	11	–	SGA	Female	Jaundice and prematurity	B3
10	18	–	–	Male	Jaundice and microcephaly	B3
11	28	–	–	Male	Jaundice and microcephaly	B1
12	21	–	–	Male	Jaundice and rash	B2
13	12	LBW	SGA	Female	Congenital heart diseases and prematurity	B1
14	18	–	–	Male	Jaundice, pneumonitis, rash, and IUGR	B3
15	7	–	–	Male	Jaundice, hepatosplenomegaly, and meningocele	B3
16	10	–	–	Female	Microcephaly and meningocele	B3
17	15	–	–	Female	Jaundice, hepatosplenomegaly, and hydrocephaly	B3
18	6	–	–	Male	Jaundice, hepatosplenomegaly, and rash	B1
19	22	–	–	Female	Hepatosplenomegaly and rash	B3
20	3	–	–	Female	Jaundice, hepatosplenomegaly, pneumonitis, and feeding difficulties	B3
21	25	LBW	SGA	Male	Prematurity and microcephaly	B1
22	7	–	–	Female	Microcephaly and IUGR	B1
23	14	–	–	Male	Hydrocephaly, pneumonitis, rash, and convulsion	B3

IUGR = intrauterine growth restriction; LBW = low birth weight; SGA = small gestational age.

**Figure 3.**
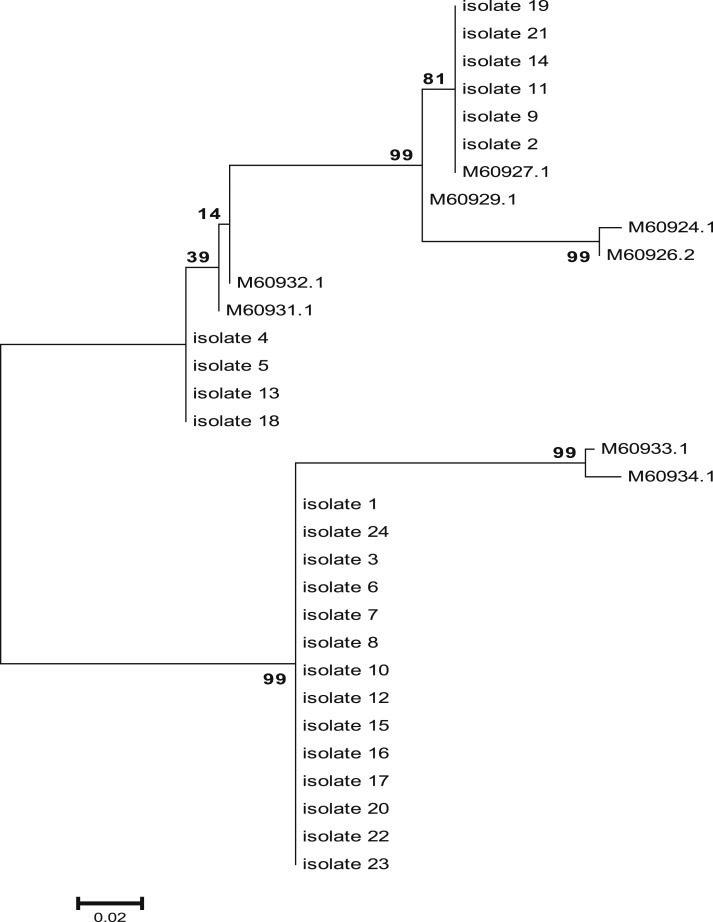
Phylogenetic tree based on gB gene constructed using the maximum likelihood method with MEGA5 software.

According to the phylogenetic tree (Figure 3), three genotypes (gB1, gB2, and gB3) were identified in our study population, whereas genotype B4 was not detected. Human cytomegalovirus gB3 was the most frequent genotype (14 [24]; 58.33%) among symptomatic infected infants, followed by gB1 (6 [24]; 25%), and gB2 (4 [24]; 16.67%). A mixed HCMV infection with gB3/gB1 was detected in only one case.

The clinical features and the genotypes of HCMV-infected neonates are shown in Table 1. No significant association (*P* > 0.05) was observed between any specific genotype and clinical feature.

## DISCUSSION

The results of the present study are comparable with the results of many global studies in which three HCMV genotypes gB1, gB2, and gB3 were identified. These three genotypes were identified for the first time in Iraq; they were also identified in samples from other parts of the world, including infected infants in China^43^ and India.^34,44^ However, four genotypes were identified in different clinical samples from children with congenital and perinatal HCMV infection in North American,^45^ Mexican,^46^ French,^35^ Dutch,^27^ and Italian children.^47^ The difference among these results may be related to the variation in HCMV genotype distribution that circulates in a population or sample.

The researchers in this study found that HCMV gB3 was the most frequent genotype among symptomatic infected infants in Iraq. A similar finding was reported in India in a study conducted on serum samples from symptomatic HCMV-infected neonates and infants.^34^ On the other hand, gB2 genotype was the most frequent genotype among HCMV-infected children, followed by gB3 in two studies conducted on urine samples in India and saliva and dried blood spot samples in Mexico.^41,46^ The gB1 genotype was the most frequent genotype in the Netherlands and Italy among HCMV-infected children, although these studies were conducted on urine samples.^27,47^ The different distribution of HCMV genotypes in congenitally and prenatally infected neonates and infants among those studies may be related to a contribution of additional factors other than geographical variation.

The prevalence of gB3 genotype in our study may reflect a different pattern of gB distribution in different clinical specimen types. Tarrago et al.^48^ have reported a significant difference in the distribution of HCMV gB genotypes among clinical sample types: gB3 was prevalent in serum samples, whereas gB2 was observed to be more predominant in cerebrospinal fluid. They conducted their work with geographically and demographically homogeneous HCMV-infected AIDS patients diagnosed with retinitis. Another study reported that gB2 and gB3 genotypes have also been associated with the expression of adhesion molecules, which may increase the spreading of these genotypes in lymphocytes.^49^ With regard to the tropism of different genotypes for peripheral leukocytes, one study reported that gB1 does not infect T lymphocytes, whereas gB2 and gB3 have the ability to infect monocytes and lymphocytes.^50^ In addition, two studies from India among congenitally and prenatally symptomatic infected HCMV neonates and infants demonstrated a different distribution of gB genotypes in serum samples^34^ and urine specimens,^41^ which supports the results of our study.

Our data indicate that HCMV gB3 was the most predominant genotype among symptomatic congenitally/perinatally HCMV-infected neonates. No association was found between gB3 and specific clinical presentation. However, more studies are required using different types of specimens to fully elucidate the prevalence of different HCMV genotypes in symptomatic neonates.
